# *In vitro* evaluation of the effect of two pediatric syrups on the microhardness and surface roughness of restoration materials

**DOI:** 10.4317/jced.62029

**Published:** 2024-09-01

**Authors:** Tania Carola Padilla-Cáceres, Heber Arbildo-Vega, Vilma Mamani-Cori, Luz Caballero-Apaza, Manuela Daishy Casa-Coila

**Affiliations:** 1 Department of General Dentistry, Dentistry School, University of the Altiplano, Puno 21001, Peru; 2 Department of General Dentistry, Dentistry School, San Martín de Porres University, Chiclayo 14012, Peru; 3 Department of Human Medicine, School of Human Medicine, San Martín de Porres University, Chiclayo 14012, Peru; 4 Department of Nursing, School of Nursing, University of the Altiplano, Puno 21001, Peru; 5 Faculty of Educational Sciences, Study Program: Science, Technology and Environment, University of the Altiplano, Puno 21001, Peru

## Abstract

**Background:**

The prolonged use of pediatric syrups without adequate control of oral hygiene can cause effects on the physical characteristics of the restoration materials, which in turn can cause deterioration of the material and subsequent carious recurrence. Aim: To evaluate the effect of two long-term use syrups in children on the microhardness and surface roughness of three restorative materials.

**Material and Methods:**

Three study groups were formed, consisting of a conventional self-curing ionomer cement, a light-curing ionomer cement, and a light-curing composite resin. Each group had 40 specimens made with the respective restorative material; in addition, these were distributed in 2 subgroups with 20 specimens each, which were immersed in Paracetamol and Ferrous Sulfate in syrup following a protocol that consisted of 2 minutes each day for 28 days.

**Results:**

Over time (0, 7, 14, 21, and 28 days), when evaluating microhardness, the composite resin subgroup exposed to ferrous sulfate (*p* = 0.027) and the Ketac Molar ionomeric cement subgroup (*p* = 0.002) exposed to Paracetamol showed statistically significant differences; while, when evaluating surface roughness, the composite resin subgroups (*p* = 0.032) and Ketac Molar ionomeric cement (*p* = 0.01) exposed to ferrous sulfate showed a statistically significant difference.

**Conclusions:**

The more days of exposure to ferrous sulphate syrup, the less the microhardness of the composite resin decreases; something similar occurs with the microhardness of Ketac Molar ionomeric cement when exposed to Paracetamol syrup. Meanwhile, the surface roughness of the composite resin and Ketac Molar ionomeric cement increases considerably when exposed to ferrous sulphate.

** Key words:**Ionomeric cement, Microhardness, Composite resins, Surface roughness.

## Introduction

When children suffer from chronic diseases, they are prescribed medications by different routes of administration; for example, the oral route is the most commonly used, through syrups, since swallowing oral medications in the form of capsules or Tablets is not practical for children (1). In order for syrups to be easily accepted by children, the pharmaceutical industry uses sucrose, which acts as a preservative and antioxidant to prolong the shelf life of the product (2,3). The solubility of some substances in syrups depends on the pH, so they are acidic preparations formulated to maintain chemical stability, optimize the efficacy of the substances and ensure optimal dispersion of the drug (1,4).

Research has shown that the use of syrup can act as an extrinsic agent for dental erosion and have negative effects on the microhardness of deciduous and permanent enamel, due to the high titraTable acidity (total acidity) and the low pH of the solution (5-7). Minimally invasive techniques using glass ionomer cements are suggested for caries management due to their effectiveness, simplicity and low cost (8), and their ability to release fluoride, providing advantages in the comprehensive treatment of children (9). The use of pediatric syrups, due to the low pH, can degrade restoration materials depending on the frequency of use (10,11). Therefore, the purpose of this research is to evaluate the microhardness and surface roughness of restoration materials subjected to prolonged use of pediatric syrups.

## Material and Methods

The study had a quantitative methodology of quasi-experimental design. To evaluate the microhardness and surface roughness, 3 dental restoration materials and 2 pediatric syrups for long-term use were selected. The selected restorative materials were: A Llis light-curing composite resin (EA2 FGM, Brazil), an I-Seal light-curing ionomeric cement (PREVEST Den Pro®, England) and a Ketac Molar self-curing ionomeric cement (3M ESPE, Seefeld, Germany). Meanwhile, the pediatric syrups selected were those that are frequently indicated by health professionals for the treatment of certain diseases; such as Paracetamol (120 mg/5 ml) indicated in cases of chronic pain, and Ferrous sulfate (75 mg/5 ml) indicated for the prevention and treatment of iron deficiency anemia.

**Figure 1:**
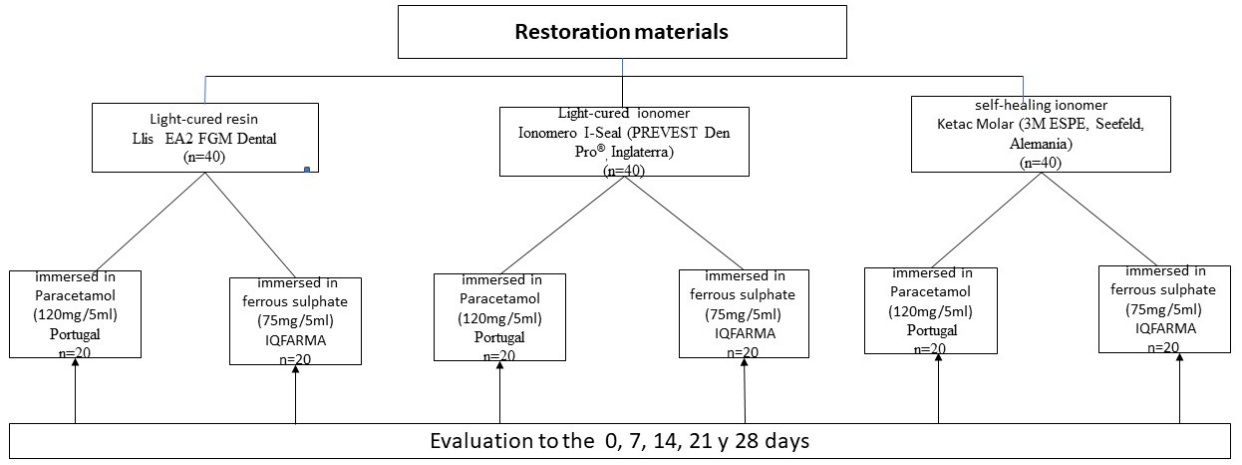
Schematic of the study groups.

Taking into consideration the restorative material, 3 study groups were formed. Each group had 40 specimens made with the respective restoration material; in addition, these were distributed in 2 subgroups with 20 specimens each, where the specimens of one of these subgroups were immersed in Paracetamol syrup (120 mg/5 ml; pH: 4.5) and the specimens of the other subgroup were immersed in Ferrous sulfate syrup (75 mg/5 ml; pH: 3.9). Adding a total of 6 study subgroups (Fig. 1).

-Preparation of specimens

Specimens were prepared in a standard plastic mold in a total of 120 disc-shaped (8 mm × 2 mm), 40 samples of each restorative material. These were prepared as described in their manufacturer’s manuals. The self-curing glass ionomer cement was allowed to set at room temperature for 10 min. The I-Seal Ionomer and Light-Curing Composite Resin were polymerized through a glass slide using a halogen light curing unit (Optilux 501, Kerr, Pomona, CA, USA) with a light intensity of 500 mW/cm2. After the procedure was completed, the samples were removed from the mold, subjected to a polishing system and then stored in deionized water at 37°C for 24 h.

-Immersion of the specimens

Specimens from the 6 study subgroups were immersed in 10 ml of solution depending on the immersion medium, Paracetamol syrup (120 mg/5 ml; pH: 4.5) or Ferrous sulphate syrup (75 mg/5 ml; pH: 3.9), for a period of 2 minutes per day with 24 hour intervals between immersion cycles. After each immersion cycle, the specimens were rinsed and stored in deionized water until the next cycle. The syrups were renewed for each immersion cycle. The microhardness and surface roughness of the restorative material were evaluated at the initial day and on days 7, 14, 21 and 28 (Fig. 1).

-Microhardness Test

The surface microhardness values were determined using a Mitutoyo HV-1000 Vickers microhardness durometer and the values obtained were recorded as Vickers hardness number (VHN) Kg/mm2. Three notches in total were made at different points of each specimen not less than 1 mm from the adjacent notch with a load of 100 gr for 15 seconds. The average of the three values obtained was recorded as VHN in) Kg/mm2.

-Surface Roughness Test

The average surface roughness (SR) values for all samples were measured with a Digital Roughness Tester (Huatec – SRT6200). Three successive measurements were recorded in different directions for each surface. Average surface roughness values were obtained. The cut-off value for surface roughness was 0.25 mm and the sampling length for each measurement was 1.5 mm. The rugosimeter was calibrated before each measurement session.

-Data analysis

Data were analyzed using the Kruskal-Wallis test for intra- and inter-group comparisons. Results were evaluated at a significance level of *p* < 0.05.

## Results

This study evaluated the microhardness and surface roughness of 3 dental restorative materials after being exposed to 2 pediatric syrups for prolonged use. In  Table 1, it can be observed that when comparing the effect of 2 pediatric syrups (Paracetamol and Ferrous Sulphate) on the microhardness of 3 restorative materials (Composite resin, I-Seal Ionomeric Cement and Ketac Molar Ionomeric Cement) over time (0, 7, 14, 21 and 28 days), the subgroup of composite resin exposed to Ferrous Sulphate and the subgroup of Ketac Molar ionomeric cement exposed to Paracetamol showed statistically significant differences (*p* = 0.027*; *p* = 0.002*), that is, in both groups as the days of exposure pass there is a decrease in the microhardness of the restorative material.

While, when comparing the effect of both syrups on the surface roughness of the 3 restorative materials, only the subgroups of composite resin (*p* = 0.032) and Ketac Molar ionomeric cement (*p* = 0.01) exposed to ferrous sulfate showed a statistically significant difference; that is, the more days of exposure to ferrous sulfate in syrup, the considerably increased the surface roughness of both restorative materials ( Table 1).

However, when comparing the effect of the 2 pediatric syrups on the microhardness of the 3 restorative materials for each time period, statistically significant differences were found for each time period (*p* < 0.05); while, when comparing the surface roughness, no statistically significant differences were found (*p* > 0.05) ( Table 1).

## Discussion

In the field of dentistry, it is very important to select the appropriate restorative materials, especially if they are to be used in the care of younger patients (11,12). Composite resins, light-curing and self-curing ionomeric cements are usually the most commonly used restorative materials in the treatment of caries lesions in temporary and young permanent dentition.

Most pediatricians and pediatric dentists have a choice for the presentation in syrups for the medications they prescribe to children, this is mainly due to the difficulty that the latter have in swallowing medications in capsule or Tablet presentations; the active compounds of the medications are essential to improve health, however, some inactive substances can be a risk for dental enamel or the restorative material (11). This study evaluated the microhardness and surface roughness of ionomeric and resinous restorative materials exposed to two syrups for prolonged pediatric use (Paracetamol and Ferrous Sulphate). Microhardness and surface roughness are important physical properties to consider in dental restorative materials (13).

Microhardness is an indicator of the material’s resistance to plastic deformation, which is related to its durability and longevity in the oral cavity; a higher microhardness of the restorative material is associated with better resistance to wear and abrasion during mastication; this is relevant, since the material must maintain its properties over time to preserve the integrity of the restoration (12,13). Meanwhile, the surface roughness of the material is crucial to avoid excessive accumulation of bacterial plaque and the development of secondary caries (12).

Regarding the superficial microhardness, it was shown that the longer the exposure time to ferrous sulphate in syrup, the greater the decrease in the microhardness of the composite resin (*p* = 0.027); Paracetamol in syrup also caused a greater decrease in the superficial microhardness in the I-Seal ionomeric cement (*p* = 0.002); both ferrous sulphate and Paracetamol showed a decrease in the microhardness values of the Ketac molar ionomeric cement at 28 days of exposure (39.4 ± 2.1; 38.1 ± 3.5), but these differences were not statistically significant (*p* = 0.613; *p* = 0.207), these results differ from those reported by Gurdogan *et al*. (10) who found a decrease in the microhardness of Ketac molar ionomeric cement when exposed to multivitamin syrups and effervescent tablets (*p*<0.05).

When evaluating the surface roughness, it was shown that a longer exposure time to ferrous sulphate in syrup caused a marked increase in the surface roughness in the composite resin (*p* = 0.032) and the Ketac molar ionomeric cement (*p* = 0.01). These results agree with Gurdogan *et al*. (10) who indicated that the Ketac molar glass ionomeric cement showed high roughness values when exposed to multivitamin syrups and effervescent tablets (*p* < 0.05). The results of this study did not show an increase in the roughness of the light-cured ionomeric cement when exposed to ferrous sulphate and paracetamol syrup (*p*=0.58; *p*=0.728), however, other studies demonstrated that the light-cured ionomeric cement presents higher values of surface roughness when exposed to agents such as probiotic mouthwash (14) and gastric acid (15).

Along the same lines, the studies by Doğu Kaya *et al*. (16) and Veček *et al*. (17) proved that the immersion of different types of resin composites and hybrid restorative cement in acidic pH drinks (green smoothie and multivitamin tablets) affected the micromechanical properties of the restorative materials such as their microhardness and roughness. Both studies showed that when exposed to acidic substances, degradation occurs and these vary considerably between materials, depending on the content of the resin compounds. Therefore, the selection of restorative materials is extremely important in patients who regularly consume acidic pH drinks, since acidic conditions can cause degradation of composite resins, due to matrix decomposition, surface erosion and dissolution (18,19). Such is the case of the consumption of Paracetamol and Ferrous Sulphate syrups, which in this study presented pH levels of 4.5 and 3.6 respectively.

Developing the evaluation of microhardness and surface roughness is essential for comparing the performance of dental restoration materials, this will allow the professional to properly determine their selection at the time of dental treatment. In this sense, it is concluded that the more days of exposure to ferrous sulphate syrup, the decrease in the microhardness of the composite resin occurs; something similar occurs with the microhardness of Ketac Molar ionomeric cement when exposed to Paracetamol syrup. Regarding the surface roughness of the restorative material, the composite resin and Ketac Molar ionomeric cement exposed to ferrous sulphate showed a considerable increase in surface roughness.

## Figures and Tables

**Table 1:** Comparison of the effect of two pediatric syrups on the microhardness and surface roughness of restorative materials.

Subgroups / Time	Initial (day 0)	7 days	14 days	21 days	28 days	p
Microhardness (Kg/mm^2^)						
Resin/Ferrous Sulphate	50.9±4.0	42±1.5	41.4±1.1	39.5±2.4	38.8±3.9	0.027*
Resin/Paracetamol	54.3±5.4	53.3±5.0	52.7±5.6	50.8±5.4	48.9±5.1	0.378*
I-Seal Ionomer/Ferrous Sulphate	19.1±3.3	10.9±1.9	10.8±1.9	10.4±2.1	10.1±2.4	0.052*
I-Seal Ionomer/Paracetamol	18.1±2.4	13.1±0.2	12.7±0.3	12.5±0.1	12±0.4	0.002*
Ketac Molar Ionomer/Ferrous Sulphate	43.1±3.1	40.6±3.3	40.4±3.7	40.1±3.4	39.4±2.1	0.613*
Ketac Molar Ionomer/Paracetamol	42.7±1.8	41.4±2.4	39.7±3.1	38.9±3.0	38.1±3.5	0.207*
p	0.001*	0.001*	0.002*	0.002*	0.003*	
Surface roughness (μm)						
Resin/Ferrous Sulphate	1.02±0.33	1.11±0.38	1.16±0.42	1.39±0.57	1.82±0.25	0.032*
Resin/Paracetamol	1.12±0.79	1.23±0.81	1.35±0.89	1.37±0.89	1.44±0.88	0.783*
I-Seal Ionomer/Ferrous Sulphate	1.22±0.38	1.27±0.39	1.38±0.42	1.44±0.37	1.55±0.36	0.580*
I-Seal Ionomer/Paracetamol	0.95±0.64	1.09±0.62	1.16±0.64	1.22±0.66	1.28±0.69	0.728*
Ketac Molar Ionomer/Ferrous Sulphate	1.49±0.12	1.61±0.12	1.68±0.09	1.72±0.08	1.83±0.08	0.01*
Ketac Molar Ionomer/Paracetamol	1.42±0.38	1.45±0.39	1.49±0.38	1.60±0.35	1.75±0.17	0.507*
p	0.387*	0.369*	0.476*	0.872*	0.565*	

*Kruskal-Wallis Test

## Data Availability

The datasets used and/or analyzed during the current study are available from the corresponding author.

## References

[1] Singana T, Suma NK (2020). An in vitro assessment of cariogenic and erosive potential of pediatric liquid medicaments on primary teeth: A comparative study. Int J Clin Pediatr Dent.

[2] Zhao D, Tsoi JKH, Wong HM, Chu CH, Matinlinna JP (2017). Paediatric over-the-counter (OTC) oral liquids can soften and erode enamel. Dent J (Basel).

[3] Mahmoud N, ELmalt M, Mohamed E (2022). Evaluation of the Erosive Effect of Pediatric Liquid Medicinal Syrups on Primary and Permanent Enamel. Al-Azhar Dental Journal for Girls.

[4] Maguire A, Baqir W, Nunn JH (2007). Are sugars-free medicines more erosive than sugars-containing medicines? An in vitro study of paediatric medicines with prolonged oral clearance used regularly and long-term by children. Int J Paediatr Dent.

[5] Fathi A, Kufiyah AK, Mohammad A, Bagasi H, Nawlalili M, Saleh Bazaid D (2021). Effect of Zamzam Water on Microhardness of Primary Tooth Enamel After Erosion Induced by Claritin Syrup: An In-vitro Study. J Int Soc Prevent Communit Dent.

[6] Scatena C, Galafassi D, Gomes-Silva JM, Borsatto MC, Serra MC (2014). In vitro erosive effect of pediatric medicines on deciduous tooth enamel. Braz Dent J.

[7] Fathi A, Kufiyah AK, Mohammad A, Bagasi H, Nawlalili M, Saleh Bazaid D (2021). Effect of Zamzam Water on Microhardness of Primary Tooth Enamel After Erosion Induced by Claritin Syrup: An In-vitro Study. J Int Soc Prevent Communit Dent.

[8] Torres PJ, Phan HT, Bojorquez AK, Garcia-Godoy F, Pinzon LM (2021). Minimally invasive techniques used for caries management in dentistry. A review. Vol. 45, Journal of Clinical Pediatric Dentistry. Journal of Clinical Pediatric Dentistry.

[9] Dhull KS, Nandlal B (2011). Effect of low-concentration daily topical fluoride application on fluoride release of giomer and compomer: An in vitro study. Journal of Indian Society of Pedodontics and Preventive Dentistry.

[10] Gurdogan Guler EB, Bayrak GD, Unsal M, Selvi Kuvvetli S (2021). Effect of pediatric multivitamin syrups and effervescent tablets on the surface microhardness and roughness of restorative materials: An in vitro study. J Dent Sci.

[11] Valera B, Bhatt R, Patel M, Patel C, Makwani D, Goyal S (2022). Effect of different pediatric medications on various tooth colored restorative materials used in pediatric dentistry. Int J Health Sci (Qassim).

[12] Ramos NBP, Felizardo KR, Berger SB, Guiraldo RD, Lopes MB (2024). Comparative study of physical-chemical properties of bioactive glass ionomer cement. Braz Dent J.

[13] de Lima Navarro MF, Pascotto RC, Borges AFS, Soares CJ, Raggio DP, Rios D (2021). Consensus on glass-ionomer cement thresholds for restorative indications. J Dent.

[14] Karatas O, Delikan E, Avunduk ATE (2024). Comparative evaluation of probiotic solutions on surface roughness and microhardness of different restorative materials and enamel. Journal of Clinical Pediatric Dentistry.

[15] İnci MA, Özer H, Özaşık HN, Koç M (2023). The effects of gastric acid on pediatric restorative materials: SEM analysis. Journal of Clinical Pediatric Dentistry.

[16] Doğu Kaya B, Yılmaz Atalı P, Özmen S, Öztürk S, Tarçın B (2024). Effect of an Effervescent Multivitamin on Color and Surface Roughness of Micro-Hybrid Dental Resin Composites. Materials.

[17] Veček NN, Par M, Sever EK, Miletić I, Krmek SJ (2022). The Effect of a Green Smoothie on Microhardness, Profile Roughness and Color Change of Dental Restorative Materials. Polymers (Basel).

[18] Soares LES, Soares ALS, De Oliveira R, Nahórny S (2016). The effects of acid erosion and remineralization on enamel and three different dental materials: FT-Raman spectroscopy and scanning electron microscopy analysis. Microsc Res Tech.

[19] Geha O, Inagaki LT, Favaro JC, González AHM, Guiraldo RD, Lopes MB (2021). Effect of Chemical Challenges on the Properties of Composite Resins. Int J Dent.

